# Exclusive Solution Discharge in Li–O_2_ Batteries?

**DOI:** 10.1021/acsenergylett.2c01711

**Published:** 2022-08-29

**Authors:** Christian Prehal, Soumyadip Mondal, Ludek Lovicar, Stefan A. Freunberger

**Affiliations:** † Department of Information Technology and Electrical Engineering, ETH Zürich, Gloriastrasse 35, 8092 Zürich, Switzerland; ‡ Institute of Science and Technology Austria (ISTA), Am Campus 1, 3400 Klosterneuburg, Austria

## Abstract

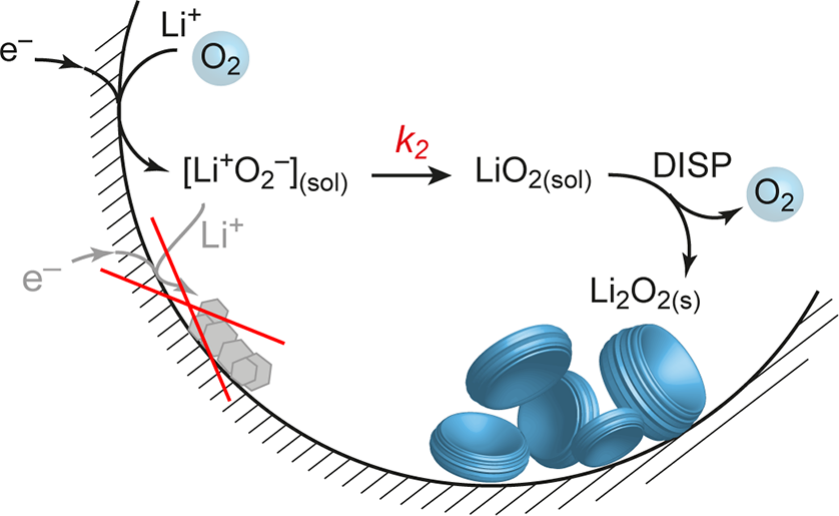

Capacity, rate performance, and cycle life of aprotic Li–O_2_ batteries critically depend on reversible electrodeposition of Li_2_O_2_. Current understanding states surface-adsorbed versus solvated LiO_2_ controls Li_2_O_2_ growth as surface film or as large particles. Herein, we show that Li_2_O_2_ forms across a wide range of electrolytes, carbons, and current densities as particles via solution-mediated LiO_2_ disproportionation, bringing into question the prevalence of any surface growth under practical conditions. We describe a unified O_2_ reduction mechanism, which can explain all found capacity relations and Li_2_O_2_ morphologies with exclusive solution discharge. Determining particle morphology and achievable capacities are species mobilities, true areal rate, and the degree of LiO_2_ association in solution. Capacity is conclusively limited by mass transport through the tortuous Li_2_O_2_ rather than electron transport through a passivating Li_2_O_2_ film. Provided that species mobilities and surface growth are high, high capacities are also achieved with weakly solvating electrolytes, which were previously considered prototypical for low capacity via surface growth.

Reducing the cost and ecological footprint of energy storage is mandatory and requires alternatives to Li-ion batteries with abundant, low-cost materials. Metal–air and metal–sulfur batteries show great potential because of the high theoretical capacities and the cheap and abundant materials.^1,2^ In both systems, insulating solids, such as Li_2_O_2_ and Li_2_S, are reversibly deposited and stripped at the cathode upon cycling. Determining the high practical capacities and lifetime are large fractions of deposited material while avoiding parasitic reactions.^2−6^ Capacity, deposit structure, and battery lifetime are intrinsically linked to the underlying physicochemical mechanisms.^5,7−10^

Current literature^2,8,11,12^ states that Li–O_2_ batteries discharge in between two limiting cases after O_2_ reduction to superoxide: (i) solution discharge, where Li_2_O_2_ forms by solution-mediated LiO_2_ disproportionation, or (ii) surface discharge, where a thin film of Li_2_O_2_ forms via direct consecutive 2 e^–^ electroreduction. Determining the predominance of a mechanism would be the current density and the electrolyte’s ability to dissociate and solvate the surface adsorbed superoxide. Solution discharge dominates in highly solvating electrolytes, enabling large (toroidal) Li_2_O_2_ particles of hundreds of nanometers and high capacities.^8,12−14^ Surface discharge is considered to dominate in weakly solvating electrolytes and at high overpotentials, leading to a passivating film and low capacities.^15−17^ Surface film growth is, in principle, self-limited by the tunneling thickness, often considered to be ∼7 nm.^16,18,19^ To what extent the surface or solution mechanism prevails is still unclear; capacity would be limited by either electron transport through a Li_2_O_2_ film or mass transport (O_2_, LiO_2_, O_2_^–^, and Li^+^) through a porous particulate Li_2_O_2_ deposit.^16,17,20−23^ In a recent study with *operando* small- and wide-angle X-ray scattering (SAXS/WAXS), we found that Li_2_O_2_ structures indicating surface growth are absent even in weakly LiO_2_-solvating electrolytes and at high overpotentials.^10^ This is in line with large Li_2_O_2_ particles imaged via electron microscopy in weakly solvating electrolytes at practical current densities and raises questions about the surface mechanism occurring.^24−27^ Consequently, truly capacity-limiting factors as well as measures and governing factors for Li_2_O_2_ packing density are still obscure.

Here we show that Li_2_O_2_ forms via solution-mediated LiO_2_ disproportionation across a wide range of relevant conditions: weakly to highly solvating electrolytes and a wide range of current densities and voltages. The obtained capacities contradict the currently accepted surface-versus-solution growth model. For instance, weakly solvating low-donor-number (DN) electrolytes, previously considered prototypical for exclusive surface growth, yield large particles and the highest capacities at low current densities. Rotating ring-disc electrode (RRDE) measurements and electron microscopy give evidence for soluble and mobile LiO_2_ even in low DN electrolytes. Supported by a numerical reaction model, we derive a Li_2_O_2_ growth mechanism that explains particle morphology and ordering across electrolytes. Capacity is limited by species (O_2_, LiO_2_, O_2_^–^, and Li^+^) transport through the porous particulate Li_2_O_2_ deposit rather than electron transport through a thin passivating Li_2_O_2_ film. The current Li–O_2_ discharge mechanism needs to be refined.

## Unexpected Performance Relations

Electrolyte solvation and applied current densities are known to significantly alter Li_2_O_2_ morphologies and achievable discharge capacities. To investigate the critical role of solvation and current densities *in conjunction*, we conducted galvanostatic discharge measurements while varying the electrolyte and carbon cathode. We used 1 M lithium bis(trifluoromethane)sulfonimide (LiTFSI) in (i) acetonitrile (MeCN), (ii) dimethylacetamide (DMAc), and (iii) tetraethylene glycol dimethyl ether containing 4000 ppm H_2_O (TEGDME/H_2_O) as electrolyte. While MeCN is weakly solvating and considered as a prototype solvent to form Li_2_O_2_ as a conformal surface coating via direct electroreduction, TEGDME/H_2_O is strongly solvating and considered to form Li_2_O_2_ as large toroidal particles via solution-mediated LiO_2_ disproportionation.^8^ The DMAc electrolyte shows intermediate solvation. We rigorously excluded H_2_O contamination since already small concentrations of H_2_O could alter product growth and discharge capacities in weakly solvating electrolytes^8^ (see Methods in the Supporting Information). To vary the current density normalized by true surface area (and overpotential), we used porous electrodes made from carbons with widely varying BET areas: glassy carbon beads (GC, 1.3 m^2^ g^–1^), Super P carbon black (SP, 55 m^2^ g^–1^), and KetjenBlack carbon black (KB, 1398 m^2^ g^–1^). An overview of current densities used in this work and literature is given in Figure S1.

**Figure 1:**
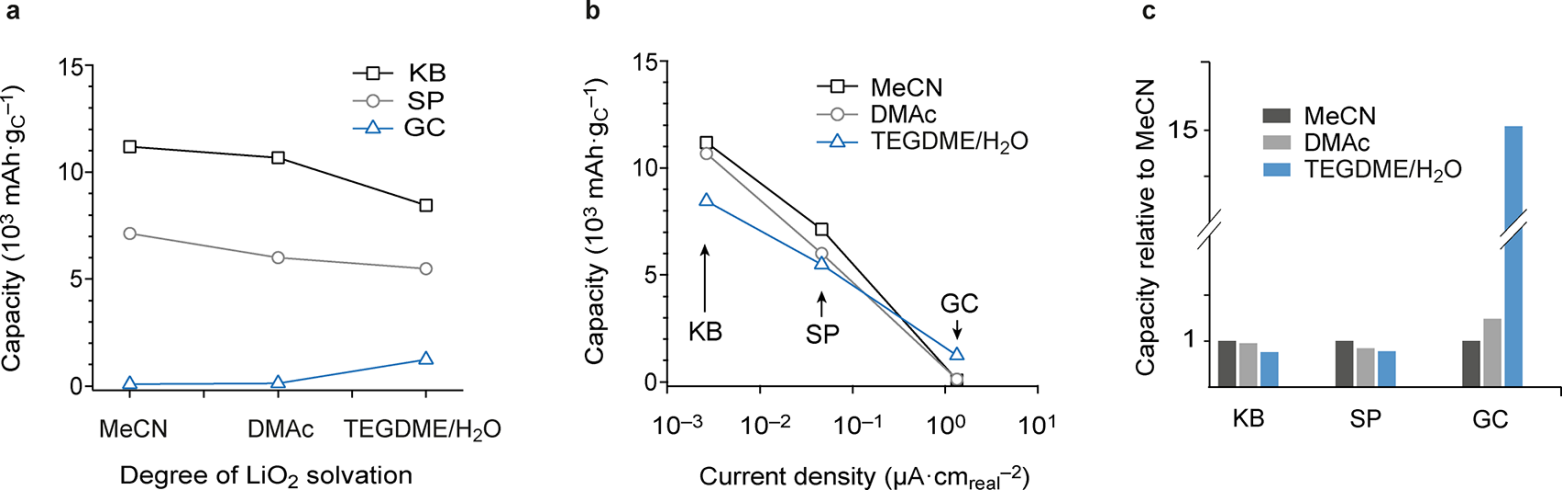
Unexpected performance relations. (a) Specific capacity versus degree of LiO_2_ solvation (governed by the electrolyte) for galvanostatic discharge at 50 μA cm_geom_^–2^. Three different carbon cathodes, KetjenBlack (KB), SuperP (SP), and glassy carbon (GC), were measured in three different electrolytes, 1 M LiTFSI in MeCN, DMAc, and TEGDME + 4000 ppm H_2_O. (b) Specific capacity versus real areal current density. Note that the order of capacity values changes systematically when going from high to low surface area carbon (KB and GC). (c) Capacity with DMAc (light gray) and TEGDME/H_2_O (blue) relative to the MeCN electrolyte (gray). Capacities have a standard deviation of ∼10% (see Figure S4).

Figure 1 presents full discharge capacities at 50 μA cm_geom_^–2^ with combinations of these three electrolytes and electrodes (Figure S2 shows cell voltage vs capacity; Table S1 summarizes normalized discharge capacities, current density, and Li_2_O_2_ degree of pore filling). Data are expressed in terms of specific capacity (mAh g_C_^–1^) as a function of LiO_2_ solvation and true areal rate (current normalized by BET area, μA cm_real_^–2^). The latter amount to 0.0027, 0.046, and 1.34 μA cm_real_^–2^ for KB, SP, and GC electrodes, respectively. Specific capacities generally increase with increasing BET area (Figure 1a) and decreasing areal rate (Figure 1b). At low and intermediate rates (with KB and SP), capacities do not follow the order of highest capacity with the highest degree of LiO_2_ solvation; the weakly solvating MeCN electrolyte gives the highest capacities, and the highly solvating TEGDME/H_2_O gives the lowest. Transition from surface to solution routes fails to explain this, suggesting that LiO_2_ solvation is not the sole factor determining capacity order at any given rate. Only the low surface area GC electrodes show the lowest capacity with MeCN and could possibly be in accord with surface growth in MeCN and successive change to solution growth in the other electrolytes.^12^ SEM images show that the Li_2_O_2_ formed at the high surface KB electrode in MeCN electrolyte to be individual, large particles of hundreds of nanometers (Figure S3). Overall, the current understanding of discharge via solution or surface routes cannot consistently explain these Li_2_O_2_ morphologies and performance relations. Solution Li_2_O_2_ growth in weakly solvating electrolytes must be considered.

## Solution Discharge in Weakly Solvating Electrolytes

**Figure 2:**
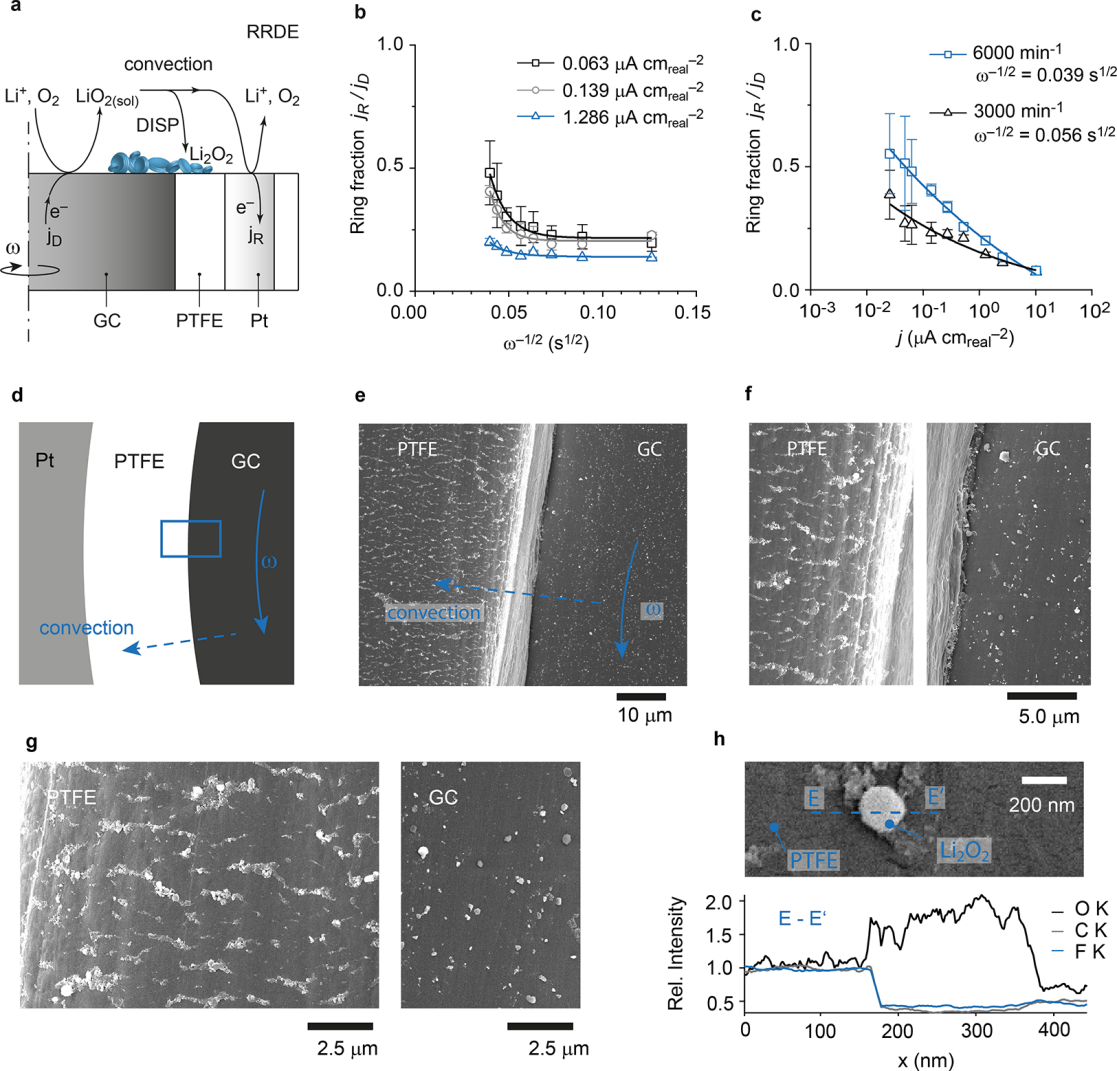
Evidence for soluble superoxide in weakly solvating MeCN electrolyte. (a–c) RRDE data with 0.1 M LiTFSI/MeCN and galvanostatic disc current. The ring was held at ∼3.6 V vs Li/Li^+^; the disc current *j*_D_ was varied between 0.025 and 10.2 μA cm_real_^–2^; the rotation rate was between 600 and 6000 min^–1^ (corresponding to ω^–1/2^ = 0.126 and 0.039 s^1/2^, respectively). The ring current, *j*_R_, is corrected for collection efficiency (*j*_R_ = −*i*_R_/*N*_0_). (a) Sketch of the RRDE and the processes. (b) The collected fraction *j*_R_/*j*_D_ as a function of rotation rate for three different disc currents *j*_D_. The solid lines are exponential fits to guide the eye. (c) The collected fraction *j*_R_/*j*_D_ as a function of disc current *j*_D_ at 3000 and 6000 min^–1^. The solid lines are power law fits to guide the eye. (d–g) SEM images of a discharged RRDE in 0.1 M LiTFSI/MeCN with *j*_D_ = 0.14 μA cm_real_^–2^ for 18 h (discharge capacity of 2.56 μAh cm_D_^–2^) at 800 min^–1^. Li_2_O_2_ particles are deposited on the GC disc and on the insulating PTFE with decaying density with growing distance from the disc edge. (h) The EDX line profile that shows that the particles on the PTFE substrate are most likely Li_2_O_2_.

Associated LiO_2_ clearly dominates in weakly solvating electrolytes, such as MeCN. Hence, solution discharge in weakly solvating electrolytes contradicts the previous understanding that associated LiO_2_ would be insoluble. To probe for soluble LiO_2_ in MeCN, we conducted RRDE measurements at true areal current densities close to those relevant for porous electrodes (discussed in Figure S1). The electrode was immersed in O_2_-flushed 0.1 M LiTFSI/MeCN electrolyte and rotated at rates ranging from 600 to 6000 min^–1^, and the ring was held at a potential where superoxide is oxidized at a transport-limited rate (Figure 2a). A constant reducing current was then applied to the GC disc in a range between 0.025 and 10 μA cm_real_^–2^. The ring current was then corrected for collection efficiency (*j*_R_ = −*i*_R_/*N*_0_) to arrive at the ring-to-disc current fraction (*j*_R_/*j*_D_), which indicates the fraction of the formed superoxide that has reached the ring electrode. The measurements go beyond previous RRDE data^10^ in that a different setup was used that allowed for higher rotation rates, improved RRDE geometry, and lower currents. Experimental details are given in Supplementary Note 1 and Figure S5.

Results in Figure 2b,c show significant ring fractions and prove that LiO_2_ is soluble in MeCN. The ring fraction increases significantly with increasing rotation rate and decreasing current and points toward a value of 1 at high rotation rates and practical current densities. The ring fraction pointing toward 1 as the transit time between disc and ring tends to zero (angular frequency ) is in accord with the solution species undergoing a chemical (C-step) but not an electrochemical reaction (E-step) during its passage from the disc to the ring. Hence, it is in accord with an EC mechanism.^28^ Ring fractions <1 cannot be explained by the partition between surface and solution mechanism as any share of the surface mechanism would be largely independent of the rotation rate. Growing ring fractions with decreasing disc current density (Figure 2c) refine the picture: while a purely homogeneous C-step would result in current-independent ring fractions, its dependence suggests a nucleation step, which is driven by high local LiO_2_ concentrations (high currents). Scanning electron microscopy of the discharged RRDE in Figure 2d–h shows that neither nucleation nor growth requires direct electroreduction (i.e., the surface mechanism) as an explanation. Particles with similar morphology as on the disc were also found on the insulating PTFE spacer. Energy dispersive X-ray measurements (EDX Figure 2h) identify them as Li_2_O_2_. RRDE and SEM data in Figure 2 give evidence for soluble LiO_2_ and solution discharge in weakly solvating electrolytes.

**Figure 3:**
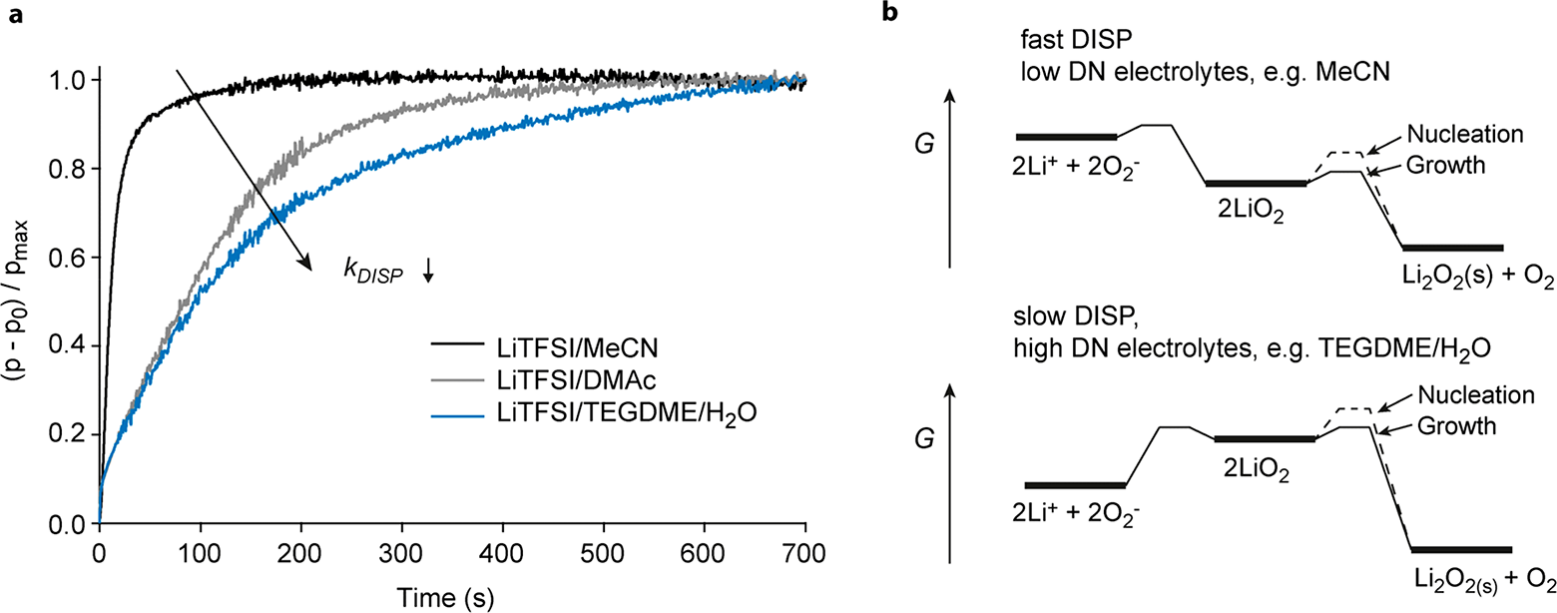
DISP kinetics in MeCN, DMAc, and TEGDME/H_2_O. (a) Pressure evolution versus time for three different electrolytes (0.1 M LiTFSI in MeCN, DMAc, or TEGDME + 4000 ppm H_2_O) upon mixing them with KO_2_ (10 mM KO_2_ in the final solution). The pressure rise stems from 2 KO_2_ + 2 Li^+^ → O_2_ + Li_2_O_2_ + 2 K^+^; its time constant is proportional to the DISP rate constant. (b) Sketch of possible free energy levels in differently solvating electrolytes during the DISP reaction in weakly solvating (low DN) and highly solvating (high DN electrolytes). The activation barrier for association in high DN electrolytes is much higher, resulting in lower DISP rate constants, lower nucleation rates, and finally fewer and larger Li_2_O_2_ particles.

High ring fractions in MeCN require small transit times between disc and ring (, Figure 2b). This suggests that the disproportionation kinetics is faster than in strongly solvating electrolytes, where soluble superoxide has already previously been identified by RRDE.^12,29^ We probed the disproportionation kinetics of KO_2_ in the three electrolytes by measuring the pressure evolution after mixing the electrolytes with KO_2_ in a custom-built pressure cell (see Methods in the Supporting Information). KO_2_ in contact with Li^+^ electrolyte disproportionates and liberates O_2_. The results in Figure 3a show that superoxide disproportionates fastest in MeCN electrolyte and slowest in the strongly solvating TEGDME/H_2_O electrolyte. This is in line with findings for NaO_2_ DISP in Na–O_2_ batteries^30^ and kinetic measurements in DMSO, MeCN, or DMF by stopped-flow UV–vis spectroscopy or SECM,^29,31,32^ but contrary to what the previous O_2_ reduction mechanism suggests: gradual shift from the surface to solution mechanism as LiO_2_ solvation decreases would imply slowing DISP in low DN electrolytes. The increased DISP kinetics in weakly solvating electrolytes show that the lower RRDE ring fractions stem from a larger fraction of the soluble LiO_2_ disproportioning to Li_2_O_2_ before it can reach the ring rather than a larger fraction of Li_2_O_2_ formed via the surface mechanism (as indicated in the sketch in Figure 2a).

We conclude that the disproportionation kinetics is related to the dissociation/association equilibrium. It defines the rate at which associated LiO_2(sol)_ feeds into the disproportionation reaction.
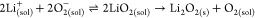
1Overall, the free energy profile of association and disproportionation may look as indicated in Figure 3b. Low barriers for growth are in accord with DFT calculations,^29^ showing that the activation barrier for disproportionation of associated LiO_2_ is low, such that its kinetics can be very fast.

## A Reconsidered Oxygen Reduction Mechanism

Previously, the partition between surface adsorbed LiO_2_* and solvated LiO_2(sol)_ (free ions, ion pairs, and clusters) has been invoked to explain a seeming shift between surface and solution growth. LiO_2_ solvation is governed by effective Lewis acidity and basicity of the electrolyte as determined by the solvent’s Gutmann donor and acceptor number (DN and AN); the salt; and, for example, protic additives.^2,11,12,14,33−35^ However, given the above presented evidence for soluble, mobile superoxide and the absence of surface growth even in weakly dissociating MeCN, the currently accepted ORR model ought to be reconsidered. Here, we describe Li_2_O_2_ formation from solution by O_2_ reduction in aprotic Li^+^ electrolytes as a function of LiO_2_ dissociation *in conjunction* with current density and LiO_2_ mobility.

In line with previous understanding, the electrolyte’s ability to solvate LiO_2_ is central for determining the Li_2_O_2_ morphology and capacity limitation. However, two modifications need to be introduced. First, LiO_2_ solvation energy comes into effect by changing the dissociation/association equilibrium in solution  and thus the rate to form associated  rather than the desorption/adsorption equilibrium between solution and surface . Second, current density and LiO_2_ mobility in the electrolyte need to be accounted for. Importantly, the new model does not contradict recent key experimental findings but revises the interpretation based on new insights. Key experimental observations are the following: (i) Capacities do not simply follow the order of highest capacity with the highest degree of LiO_2_ dissociation at all current densities (Figure 1). (ii) LiO_2_ is soluble and mobile even in weakly solvating electrolytes (Figure 2). (iii) Li_2_O_2_ forms to the widest extent via solution-mediated DISP (Figure 2 and a recent *operando* SAXS/WAXS study^10^). (iv) Li_2_O_2_ particles become smaller and more numerous with increasing current (*operando* SAXS/WAXS^10^ and refs (8),^14^, (36), and (37)). (v) Li_2_O_2_ particles become larger and less numerous with increasing LiO_2_ dissociation (*operando* SAXS/WAXS^10^ and refs (8), (12), and (14)). (vi) Weakly solvating electrolytes accelerate superoxide disproportionation rather than slowing it down (Figure 3, refs (29) and (32)).

**Figure 4:**
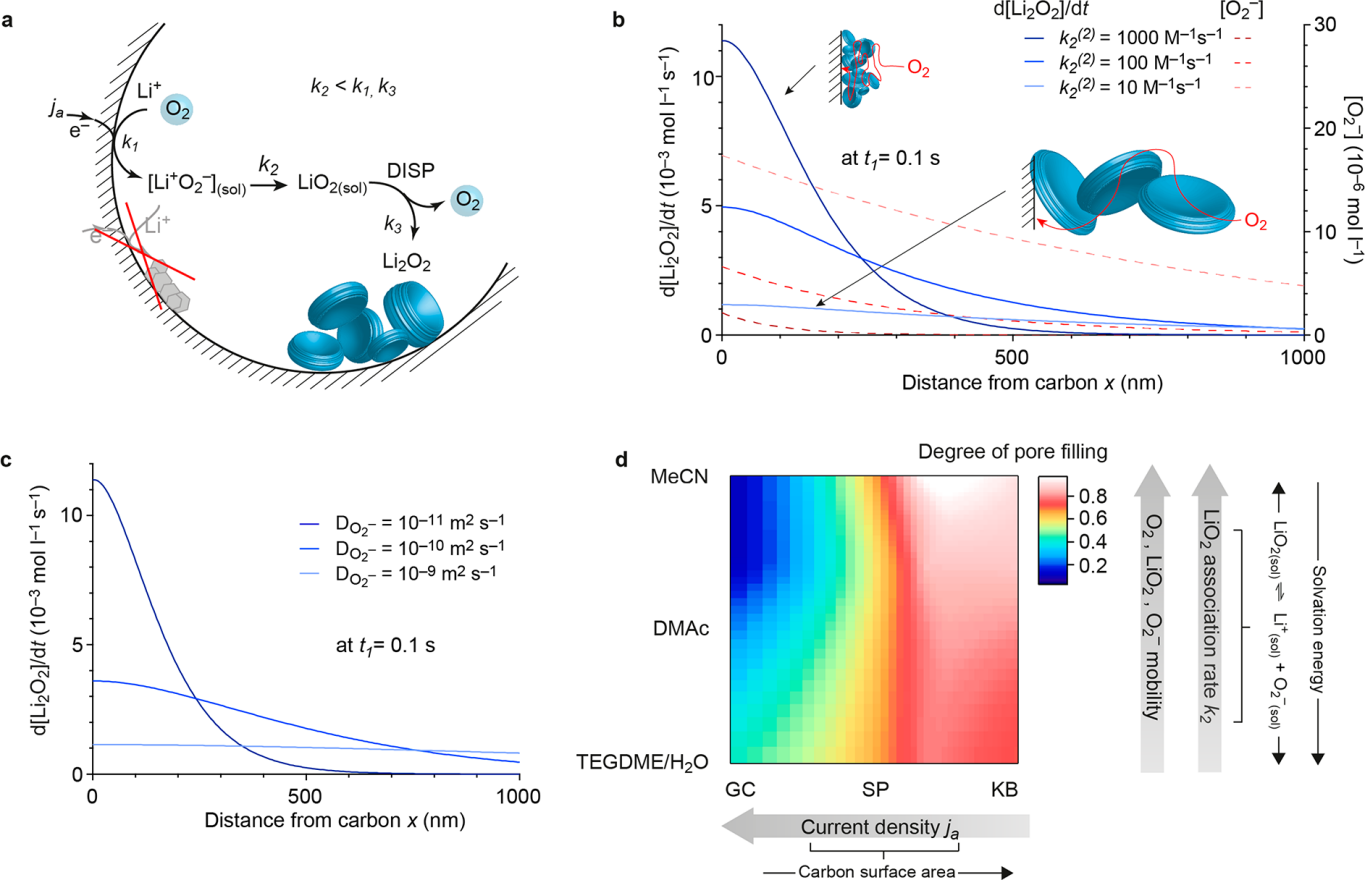
Li_2_O_2_ growth model and governing factors for morphology and pore filling. (a) Sketch of oxygen reduction and Li_2_O_2_ formation mechanism and morphology. (b) Li_2_O_2_ formation rate and O_2_^–^ concentration versus normal distance from the carbon surface as obtained from a numerical model. The example shows the impact of electrolyte solvation and thus the association kinetics *k*_2_. Fast association (high *k*_2_, dark blue curve) causes fast Li_2_O_2_ formation close to the surface and steep O_2_^–^ concentration gradients, leading to high near-surface nucleation rates and a large number of small particles. Slow association (low *k*_2_, light blue curve) results in few, larger particles up to larger distances. (c) Li_2_O_2_ formation rate profiles for different O_2_^–^ diffusivities. Lower diffusivities result in high rates of Li_2_O_2_ formation close to the surface and a high density of small, near-surface Li_2_O_2_ particles. The impact of current densities and the time dependency is explored in Supplementary Note 2. (d) Degree of pore filling with Li_2_O_2_ calculated from capacities in Figure 1. Note that the apparent high degree of pore filling (close to one) can be explained only by significant electrode swelling, as discussed in Supplementary Note 4. Arrows indicate factors influencing the Li_2_O_2_ morphology, pore filling and discharge capacity.

Deciding for Li_2_O_2_ formation is the association of solvated LiO_2_ according to the equilibrium . LiO_2(sol)_ denotes associated species such as contact ion pairs or clusters as typical for ionic species in aprotic media.^12,33,38^ This equilibrium defines the rate at which associated LiO_2(sol)_ feeds into the disproportionation reaction with the overall sequence
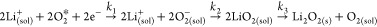
2Note that eq 2 may involve an additional LiO_2(sol)_ adsorption step prior to disproportionation, as physi- or chemisorbed LiO_2_ on existing Li_2_O_2_ crystallites has been ascertained experimentally.^39^ The actual disproportionation step (*k*_3_) of chemisorbed LiO_2_ might even occur in the solid state. Electrolyte and current density dependence of the process in eq 2 and capacity limitations are illustrated in Figure 4 and discussed in the following.

Superoxide forms at a rate proportional to the current density *j*_a_ and associates with Li^+^ with the rate constant *k*_2_ to LiO_2(sol)_, which then disproportionates with the rate constant *k*_3_ to Li_2_O_2_ and O_2_ (Figure 4a). Since superoxide disproportionation passes via the (LiO_2_)_2_ dimer or higher aggregates,^29,38,40^ its formation from 2 LiO_2(sol)_ is strongly favored over formation from 2 Li^+^_(sol)_ + 2 O_2(sol)_^–^. Disproportionation of associated LiO_2(sol)_ is second order in LiO_2(sol)_ concentration and very small activation barriers suggest *k*_3_ to be very large. Superoxide disproportionation all the way from O_2_^–^ to Li_2_O_2_ can be regarded as a pseudo-first order reaction in O_2_^–^ since the Li^+^ concentration is orders of magnitude higher than the O_2_^–^ concentration.^29,31^ Association is hence the rate-limiting step in eq 2 and determines the overall rate to form Li_2_O_2_ via disproportionation. The association rate constant *k*_2_ depends on the solvation strength of the electrolyte and is connected with the dissociation/association equilibrium (see eq 1 and Figure 3).

Low solvation energies (weakly dissociating electrolytes) shift the dissociation equilibrium toward associated LiO_2(sol)_, in turn increasing the association rate constant *k*_2_ (Figure 3). Figure 4a and eq 2 illustrate that the profile of O_2_^–^ concentration versus distance from the electrode surface determines local Li_2_O_2_ nucleation and growth and hence particle density and size. The Li_2_O_2_ formation rate profile arises from solvation, current density, and species mobility in conjunction.

To better grasp the mutual sensitivity of electrolyte solvation (LiO_2_ association), true areal current densities, and species mobilities, we implemented a simple 1D numerical model taking into account O_2_^–^ production at the electrode interface, diffusive transport away, and disproportionation as a sink with a rate governed by the O_2_^–^ concentration profile. The model intends to identify the important trends rather than accurately accounting for (heterogeneous) nucleation and growth of Li_2_O_2_ particles or the real carbon electrode structure. Further details and results are given in Supplementary Note 2, Table S2, and Figures S6 and 4b,c.

The model is based on eq 2 and a pseudo-first order DISP kinetics with respect to O_2_^–^ concentration as revealed by stopped-flow UV–vis spectroscopy.^29,31^ Considering eq 2 and the fact that LiO_2_ association is rate-limiting (*k*_2_), DISP at a planar electrode can be modeled by the following process:
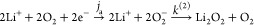
3Herein,  is the second-order DISP rate constant with respect to  and , which translates into the pseudo-first order DISP rate constant  with respect to . The model calculates the concentration profile  and  as a function of distance *x* from a planar electrode surface and time *t* by solving the following partial differential equations numerically via a finite difference method^41^ assuming constant current
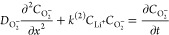
4
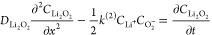
5Equations 4 and 5 account for the diffusion of O_2_^–^ and Li_2_O_2_ via Fick’s law. O_2_^–^ consumption and Li_2_O_2_ generation are considered as a sink term (eq 4) and source term (eq 5). The sink term is expressed by the second-order reaction ν = , or the equivalent pseudo-first order reaction ν = .

The resulting Li_2_O_2_ concentration profile  (Figure S6d–f) gives an estimate for the thickness of the particulate Li_2_O_2_ layer on the electrode surface and the local rate at which Li_2_O_2_ forms. A high  means a large quantity of Li_2_O_2_ formed at a high rate. We calculate the local Li_2_O_2_ formation rate  by dividing  by the time step . A high local Li_2_O_2_ formation rate causes high nucleation rates. Li_2_O_2_ particles would be smaller and more numerous.

Considering first the effect of LiO_2_ solvation (Figure 4b), weakly dissociating electrolytes (i.e., large *k*_2_) cause high O_2_^–^ concentration and Li_2_O_2_ formation rates close to the electrode surface, and both sharply decay as distance grows. The used reaction rates (*k*_2_) are in the range of experimental values (disproportionation rate constants of MeCN electrolyte,^31^ 25 s^–1^; DMSO electrolyte,^29^ 560 s^–1^). High near-surface Li_2_O_2_ formation enhances near-surface nucleation, causing a larger number of small particles closer to the surface. Highly dissociating electrolytes with slow association kinetics *k*_2_ cause low Li_2_O_2_ formation rates at all distances from the electrode surface and flat O_2_^–^ concentration gradients. This leads to low nucleation rates and few large Li_2_O_2_ particles that reach out several 100 nm into solution, in line with literature and our recent *operando* SAXS study.^10^ To confirm this with mainly varying association while leaving transport largely constant, we tested dimethoxyethane (DME) electrolytes and different LiTFSI/LiNO_3_ concentrations ratios (Supplementary Note 3 and Figures S7 and S8). Adding NO_3_^–^ significantly increases LiO_2_ dissociation^42,17^ but does not primarily affect Li^+^, O_2_, and LiO_2(sol)_ diffusion coefficients.

As visualized by the sketches in Figure 4b, the increasingly tortuous transport path self-accelerates tortuosity increase with growing depth of discharge, finally causing the end of discharge by mass transport limitation (O_2_, Li^+^) toward the electrode surface combined with some degree of surface blocking by Li_2_O_2_ particles touching the carbon.^21^ Dominance of surface growth even in MeCN implies these factors are limiting in all electrolytes.

Considering further superoxide mobility and current density, both in conjunction determine the near-surface O_2_^–^ concentration and Li_2_O_2_ formation profile, in turn Li_2_O_2_ particle density/size, and how far the layer of Li_2_O_2_ particles can reach out from the surface (Figure 4c). This layer thickness determines the achievable degree of pore filling and hence capacity. Growing currents in a certain electrolyte cause growing O_2_^–^ concentrations and steeper gradients, as illustrated in Supplementary Note 2 and Figure S6. Higher Li_2_O_2_ formation rates close to the carbon surface enhance near-surface nucleation, causing a larger number of small particles closer to the surface compared to low current.

With these relations between LiO_2_ dissociation, species mobility, and true surface current density in mind, a map of achievable capacity can be drawn as illustrated in Figure 4d. It is in accord with the capacities in Figure 1 from where the degree of pore filling is taken for the contour plot. Importantly, Li_2_O_2_ particle size alone determines discharge capacities only at planar/low surface area electrodes. In moderate to high-surface area cathodes (where the pore size ≈ Li_2_O_2_ particle size), pore filling does. Next to the (i) LiO_2_ association rate, the other main parameters are (ii) current (raising local superoxide concentration and hence nucleation) and (iii) superoxide and other species mobilities^43,44^ (determining how far LiO_2(sol)_ can diffuse before it disproportionates and how tortuous the Li_2_O_2_ deposit may be before causing mass transport limitations). High disproportionation rates in weakly dissociating electrolytes are not detrimental if (i) areal current densities are low and (ii) species mobilities are high. The highest capacity being achieved with MeCN electrolyte and KB electrode even at high geometric rates confirms this (additional data and discussion in Supplementary Note 5 and Figure S9). To give absolute numbers of required current densities or species mobilities, future model calculations need to consider the actual porous electrode structure and the increasing tortuousity caused by Li_2_O_2_ formation.

By combining galvanostatic discharge with RRDE measurements, electron microscopy, O_2_ pressure evolution measurements, and a 1D numerical model, we show that discharge of aprotic Li–O_2_ batteries proceeds to the widest extent via solution-mediated LiO_2_ disproportionation to form Li_2_O_2_ particles. Li_2_O_2_ forming a passivating film via direct electroreduction of surface adsorbed LiO_2_ can be largely excluded under practically relevant conditions. This is true even for low DN electrolytes previously considered prototypical for the surface mechanism. Species transport through the increasingly tortuous particulate Li_2_O_2_ deposit hence limits capacity rather than electron transport across Li_2_O_2_ films. We describe a unified O_2_ reaction mechanism that can explain Li_2_O_2_ particle size and number density, packing density, achievable rate, and capacity across a wide range of electrolytes and operating conditions. Deciding factors are the dissociation of solvated LiO_2_, species mobilities (Li^+^, O_2_, O_2_^–^, and LiO_2_), and areal current densities.

This mechanism suggests strategies on how research toward highly reversible, high-performance Li–O_2_ cells should proceed. First, low-donor-number (weakly LiO_2_ dissociating) electrolytes, previously thought to be prototypical for low capacity, can achieve the highest pore filling and capacity. High species mobility and high electrode surface area are, however, a requirement. Mediators, for example, make superoxide more mobile^45,46^ and allow oxidizing large particles and suppressing side reactions,^47^ but their impact on, for example, packing density and ordering remains to be studied. They also shift the O_2_ reduction from the surface to the electrolyte volume,^48^ reduce high near-surface nucleation, and may hence allow for lower-surface electrodes to achieve large capacities. Second, the previous paradigm can be lifted that highly solvating electrolytes are required for high capacity despite them being more susceptible to decomposition. Disproportionation has, however, been shown to be the source of the highly reactive singlet oxygen, which in turn is the major source of parasitic reactions and requires careful consideration when judging electrolytes.^5^ We further note that the here derived mechanism holds for relatively defect-free carbon surfaces as found with pristine GC, SP, and KB, where LiO_2_ adsorbs weakly.^49^ Highly defective carbonaceous electrodes^49^ or catalyst surfaces^50^ could change LiO_2_ adsorption and rates and hence favor the surface mechanism to some extent.

The current picture of Li_2_O_2_ formation, proceeding in-between the two cases of surface and solution mechanism, ought to be reconsidered. Why the second consecutive electron transfer at the carbon surface mechanism is so unlikely compared to LiO_2_ disproportionation remains to be clarified.

## Supporting Information Available

Methods, materials, and experimental procedures; Supplementary Figures S1–S10, Supplementary Tables S1 and S2, and Supplementary Notes 1–5 (PDF)
