# Supplementary material for: Exclusive Solution Discharge in Li–O2 Batteries?
Source: ACS Energy Lett. 2022 Aug 29;7(9):3112–9. doi: 10.1021/acsenergylett.2c01711 (PMC9469202; doi:10.1021/acsenergylett.2c01711)
Supplement: Supplementary file 1 — nz2c01711_si_001.pdf [file nz2c01711_si_001.pdf]

Supplementary Information

Exclusive solution discharge in Li-O_2 batteries?

Christian Prehal[1,*], Soumyadip Mondal[2], Ludek Lovicar[2], Stefan A. Freunberger[2,*]

1. Department of Information Technology and Electrical Engineering, ETH Zürich, Gloriastrasse 35, 8092 Zürich, Switzerland
2. Institute of Science and Technology Austria (ISTA), Am Campus 1, 3400 Klosterneuburg, Austria

* Corresponding author's e-mail: cprehal@ethz.ch; stefan.freunberger@ist.ac.at

1. Methods

Materials

Lithium bis(trifluoromethane)sulfonimide (LiTFSI) from SOLVIONIC was dried under reduced pressure for 24 h at 140°C. MeCN and DME were distilled under Ar over CaH_2. DMAc and TEGDME were distilled under vacuum. All solvents were further dried and stored over freshly activated molecular sieves (type 4 Å).

The water content of the electrolytes was determined by Karl-Fischer titration using a Methrom 851 Titrando and found to be ≤ 30 ppm for all electrolytes. For the MeCN solvent, Karl-Fischer titration revealed a water content of < 6ppm. Adding 1 M of LiTFSI raised the water content by roughly 10 ppm. After hours in direct contact with O_2 the water content of MeCN was still found to be 6.2 ppm. The electrolyte used in the RRDE measurements was measured after the experiments and found to contain a maximum of 11 ppm H_2O.

The BET areas of glassy carbon spherical powder (Aldrich), Super P (Timcal), and KetjenBlack (AkzoNobel) were determined by N_2 gas adsorption and found to be 1.3 m^2g^{-1}, 55 m^2g^{-1} and 1398 m^2g^{-1}, respectively. Electrodes were made by mixing carbon with polytetrafluoroethylene (PTFE, 60 mass% suspension in water, Aldrich) at 90/10 (w/w) ratio with isopropanol. The resulting dough-like material was rolled to a 50-70 µm thick free standing film electrode, washed in acetone/H_2O mixture and finally dried at 120 °C under vacuum overnight. Specific surface areas, specific pore volumes, electrode densities, and total porosities of the three carbon electrodes are given in Table S2. Partially delithiated Lithium iron phosphate (LFP) was used for the counter electrode material which was made by mixing LFP, delithiated LFP, Super P, and PTFE in a 62/17/11/10 (w/w) ratio with isopropanol and rolling resulting material to a 500 µm thick free standing film electrode, washing in acetone/H_2O mixture and drying at 120 °C under vacuum overnight. All electrodes were transferred to an Ar-filled glovebox without air exposure.

Experimental

Galvanostatic discharge measurements were conducted in standard laboratory Li-O_2 cells using a MPG-2 galvanostat (Biologic). The cells used a sandwich of carbon cathode (50-70 µm thick, 8mm in diameter), an electrolyte soaked Whatman GF/A separator, and an oversized partially delithiated $Li_{1-x}FePO_4$ (MTI corporation) counter electrode (500 µm thick, 12 mm in diameter). Al grid current collectors were used to ensure continuous O_2 supply.

For the scanning electron microscopy (SEM) measurements a FEG-SEM Carl Zeiss MERLIN VP Compact was used. Prior to the measurements, Pt was sputtered to minimize the electron beam induced damage and to avoid charging at the PTFE. A high vacuum sputtering system Leica EM ACE600 was used to deposit 1.6 nm Pt. To ensure high purity of the Pt layer, the sputtering system was purged three times with Ar gas prior to sputtering. The SEM signal was detected using an in-column in-lens secondary electron detector and by in-chamber HE-SE2 Everhart-Thornley detector. The Pt layer allowed to use 5 kV accelerating voltage in combination with a 10 µm aperture. Higher accelerating voltage increased the contrast between the surface and the crystals, improving subsequent data analysis. Energy Dispersive Spectroscopy (EDS) was acquired using an EDAX Octane Elite Super 70mm^2 controlled by APEX 1.5 Advanced Software and equipped with a SiN window.

Rotating ring disc electrode (RRDE) measurement were performed inside an Ar-filled glovebox using a BluRev rotator and an RRDE with 3 mm GC disc and a Pt ring with 4 (6) mm inner (outer) diameter (Biologic, France). A SP-300 potentiostat/galvanostat (Biologic, France) with ultra-low current cell cables was used for the measurements. Measurements were performed in a cylindrical glass cell with narrow upper opening through which the shaft, an Ag wire as reference electrode, $Li_{1-x}FePO_4$ pasted on a stainless steel grid as counter electrode, and a tube for O_2 were inserted. After O_2 saturation by direct

bubbling, the electrolyte was further bubbled during the entire measurement. The electrode was polished before every experiment using 0.05 mm alumina slurry in isopropanol, rinsed with MeCN and dried under vacuum. 2 mM ferrocene in 0.1 M TBATFSI in MeCN were used to determine the collection efficiency N_0 of the ring. Disproportionation kinetics of KO_2 in various electrolytes was measured by placing KO_2 powder in a closed reaction vessel equipped with a pressure sensor (Omega, PAA35X) as described previously[6] and injecting the electrolyte using a syringe through a septum.

2. Supplementary Figures

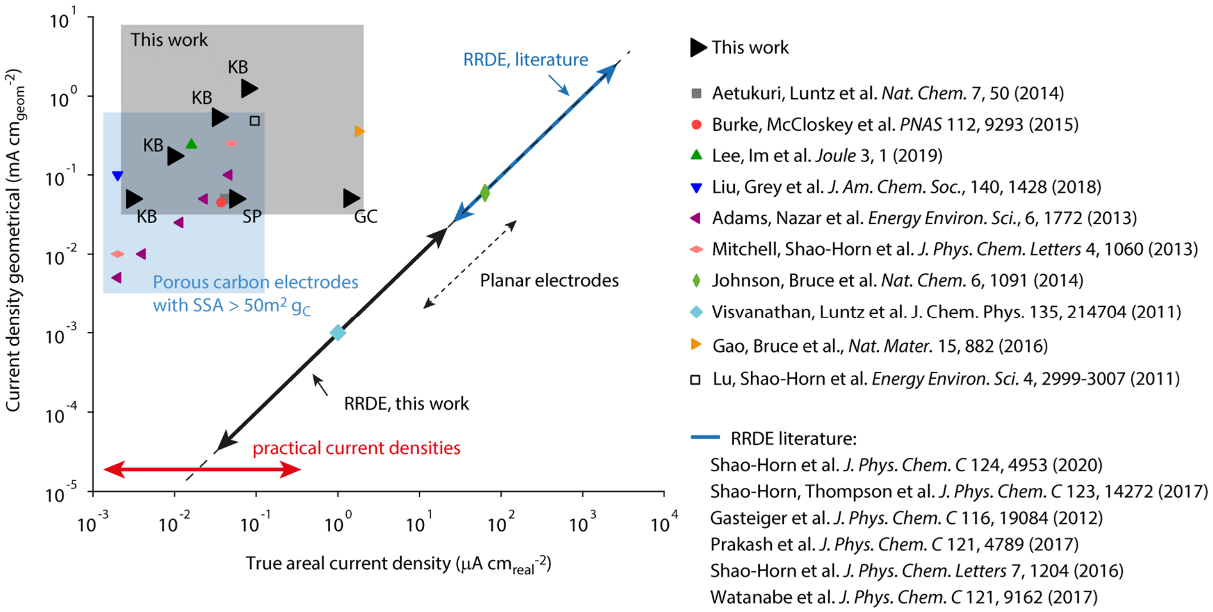

Figure S1. Geometric vs. true areal current densities used in this work and in literature. Current density geometrical (normalized by the electrode area, which is 0.5 cm^2) vs. true areal current density (normalized by the specific surface area, SSA or the BET area) of literature values and measurements in this work. Only those literature values were taken that still allowed for significant capacities (> 2000 mAh g_C^{-1}). Practical true areal current densities allowing significant capacities in porous carbon electrodes (with BET areas ranging from 1 – 1500 m^2 g_C^{-1}) range from about 0.001 to about 0.2 µA cm^{-2}.

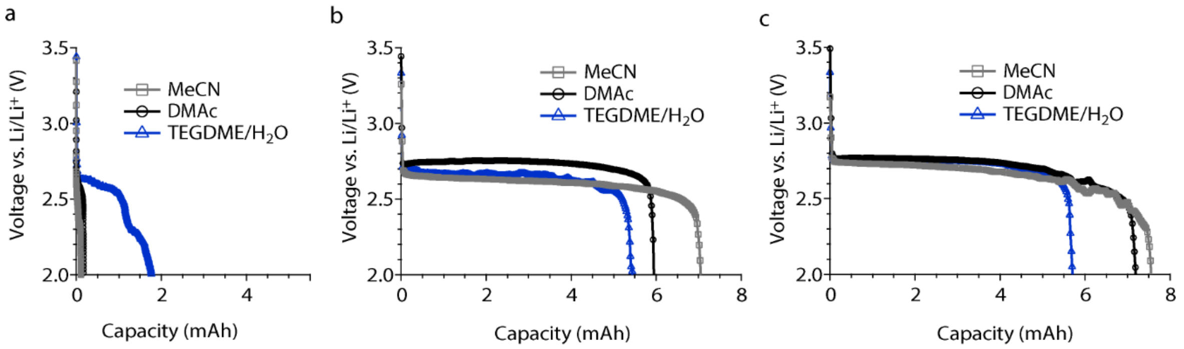

Figure S2. Galvanostatic discharge – standard cells. (a)–(c) Voltage versus capacity for galvanostatic discharge at 50 µA cm^{-2} using GC (a), SP (b) and KB (c) cathodes in standard electrochemical Li-O_2 cells.

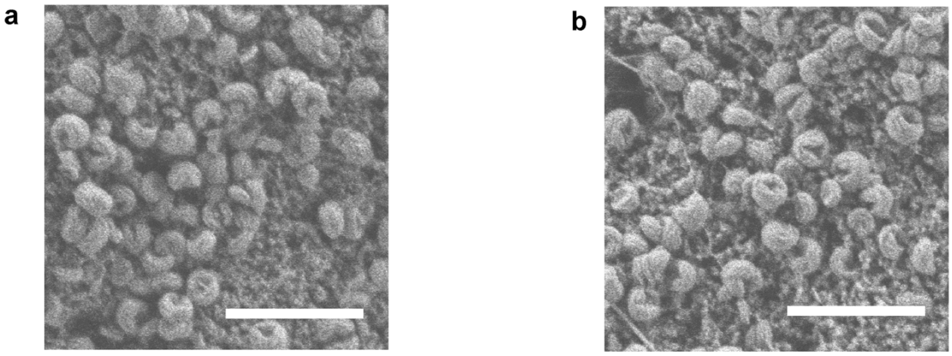

Figure S3. SEM micrographs of a galvanostatically discharged KB electrode (180 µA cm_{geom}^{-2}) in 1 M LiTFSI in MeCN at a capacity of 1380 mAh g_C^{-1}. (a) Electrode side facing the O_2 reservoir. (b) Electrode side facing the separator. Scalebar = 1 µm.

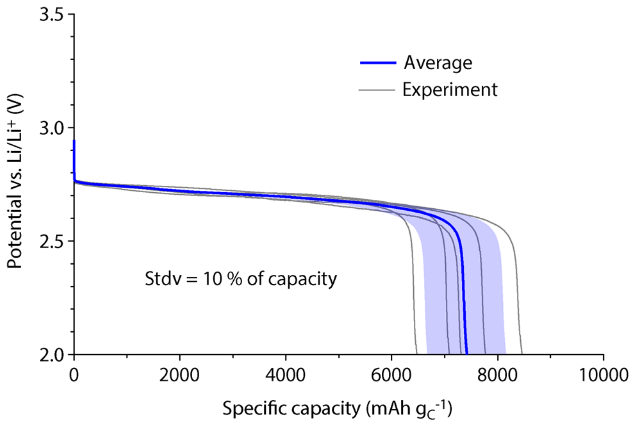

Figure S4. Reproducibility test of galvanostatic discharge. Galvanostatic discharge at 0.5 mA cm^{-2} (current normalized by electrode area, 0.5 cm^2) of five KB electrodes in a 1 M LiTFSI / MeCN electrolyte. The blue curve represent the mean discharge curve, the blue shaded region the standard deviation, amounting to 10% of the discharge capacity. Sources of error are the imprecise weighing of electrodes and some variation in handling during assembling the Swagelok cells.

3. Supplementary Tables

Table S1. Capacities, normalized discharge capacities, current density, and Li_2O_2 degree of pore filling for galvanostatic discharge measurements at 50 μAcm_{geom}^{-2}.

	Capacity (mAh)	Specific capacity ($mAh \cdot g_C^{-1}$)	Capacity per electrode area ($mAh \cdot cm^{-2}$)	Capacity per BET area ($\mu Ah \cdot cm^{-2}$)	Capacity per pore volume ($mAh \cdot cm^{-3}$)	Current density ($\mu A \cdot m^{-2}$)	Li_2O_2 pore filling
KB + MeCN	7.6	11200.00	15.04	0.80	2605.64	26.50	0.945
SP + MeCN	7.1	7127.27	14.04	13.08	2904.81	463.35	1.054
GC + MeCN	0.1	78.89	0.23	6.07	74.16	13354.70	0.027
KB + TEGDME/H_2O	5.7	8462.22	11.36	0.61	1968.70	26.50	0.714
SP + TEGDME/H_2O	5.4	5484.85	10.80	10.06	2235.42	463.35	0.811
GC + TEGDME/H_2O	1.8	1241.67	3.56	95.51	1167.31	13354.70	0.423
KB + DMAc	7.2	10674.07	14.33	0.76	2483.28	26.50	0.901
SP + DMAc	6.0	6006.06	11.83	11.02	2447.84	463.35	0.888
GC + DMAc	0.2	117.15	0.34	9.01	110.14	13354.70	0.040

4. Supplementary Notes

Supplementary Note 1: Rotating Ring Disc Electrode (RRDE) measurements

Figure S5a,c show the background subtraction procedures during RRDE measurements; Figure S5b,d ring current, disc current and disc potential of the corresponding measurements.

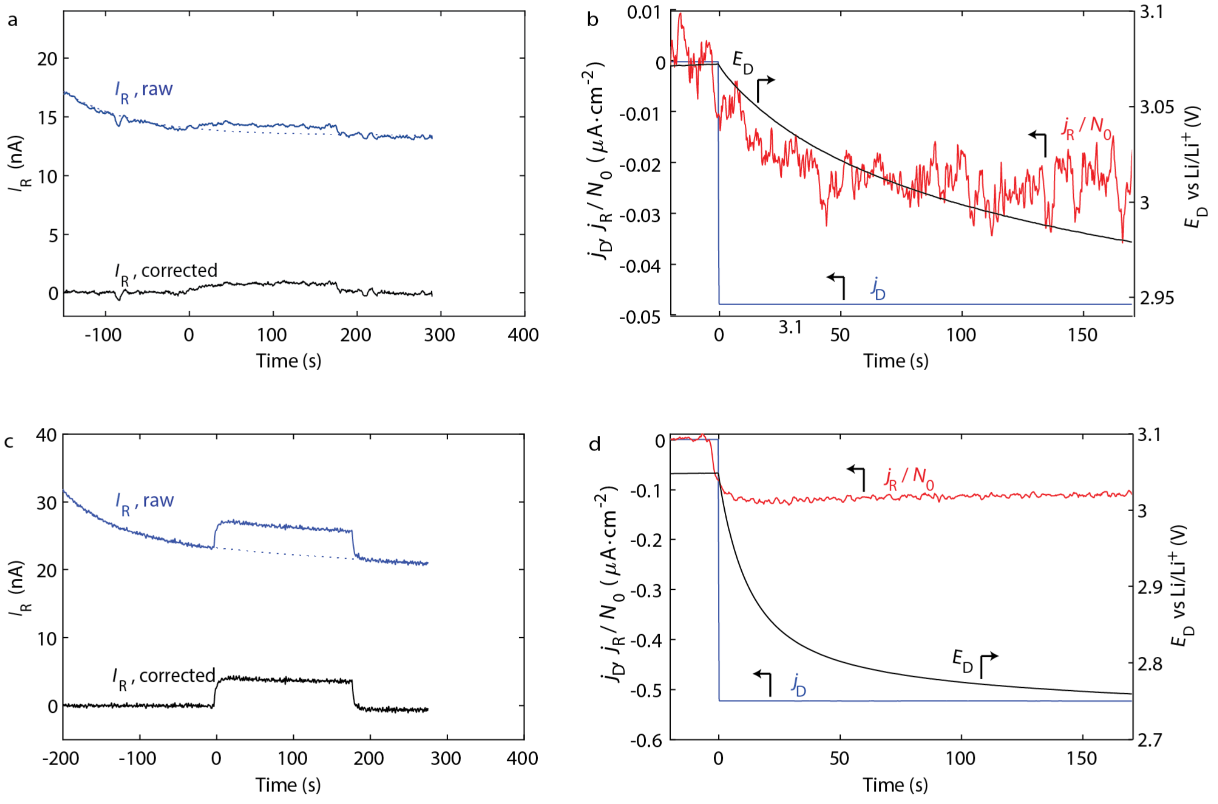

Figure S5. RRDE measurements with small currents at the disc (Figure 2). The ring potential was swept at 100 mV s^{-1} from its OCV to ≈3.6 V vs Li/Li$^+$ and held thereafter After 5 min, when the measured ring current $i_{R,raw}$ (blue trace in (a) and (c)) was nearly constant, the disc current was switched on for 3 min and off thereafter to obtain again the background value. Since the ring background current is non-negligible, the background was fitted using $i_{\text{background}}(t) = i_0 \cdot \exp(-k(t-t_0)) + k_l t + i_b$ (dotted trace in (a) and (c)) and subtracted to obtain the background corrected ring current $i_{\text{R,corrected}}$ (black trace in (a) and (c)) which is used in Fig. 4b,c. The time axis is relative to the start of the disc current. The examples show (a, b) disc current i_D = 3.35 nA (j_D = 0.048 µA cm$_{real}^{-2}$) at a rotation rate of 6000 min^{-1} and (c, d) i_D = 36.8 nA (j_D = 0.52 µA cm$_{real}^{-2}$) at a rotation rate of 3000 min^{-1}.

Supplementary Note 2: Model calculations for superoxide diffusion and disproportionation rates

We have implemented a simple numerical model to calculate O_2^- concentration, Li_2O_2 concentration and Li_2O_2 formation rate profiles at a given current density, disproportionation rate constant (meaning association k_2 in Eq. 2) and O_2^- diffusivity. Despite its simplicity, the model correctly predicts morphology (and capacity) relations over the entire range of current densities, species mobilities, and electrolyte solvation strengths. The model intents to grasp the mutual sensitivity of Li_2O_2 formation on these three parameters rather than to quantitatively merge experimental observations with the model output.

The model is based on equation (2) in the main text and a pseudo-first order disproportionation (DISP) kinetics with respect to O_2^- concentration as revealed by stopped-flow UV-vis spectroscopy[2,3].

The association of O_2^- and Li^+ is a pseudo-first order reaction in O_2^-, since the Li^+ concentration is much higher than the local O_2^- concentration. This indicates that the rate-limiting step is the association of Li^+ and O_2^- to form LiO_2: $\mathrm{Li}^+_{(\mathrm{sol})} + \mathrm{O}^-_{2(\mathrm{sol})} \rightarrow \mathrm{LiO}_{2(\mathrm{sol})}$. If the subsequent disproportionation step $2\mathrm{LiO}_{2(\mathrm{sol})} \rightarrow \mathrm{Li}_2\mathrm{O}_2 + \mathrm{O}_2$ were rate-limiting, the experimentally determined reaction rate would have to be of second order or higher since at the least 2 $LiO_{2(sol)}$ need to form $(LiO_2)_{2(sol)}$ dimers or higher aggregates.

According to equation (2) in the main text, superoxide forms at a rate proportional to the current density j_a and associates with Li^+ with the rate constant k_2 to $LiO_{2(sol)}$, which then disproportionates with the rate constant k_3 to Li_2O_2 and O_2. Since the k_2 is rate-limiting and k_3 is sufficiently fast, DISP at a planar electrode can be modelled by the process in Eq. 3 in the main text. The concentration profiles $C_{\mathrm{Li}^+}(x,t)$ and $C_{\mathrm{O}_2^-}(x,t)$ are governed by the partial differential equations Eq. 4 and 5.

Peng et al. [Ref.[2]] have measured a reaction rate of $k^{(2)} = 25\ \mathrm{M}^{-1}\mathrm{s}^{-1}$ in a DMSO solution, which translates to a pseudo-first order reaction rate of $k^{(1)} = 25\ s^{-1}$ at 1 M Li^+ concentration. For MeCN they obtained of $k^{(2)} = 560\ \mathrm{M}^{-1}\mathrm{s}^{-1}$, which translates to a pseudo-first order reaction rate of $k^{(1)} = 560\ s^{-1}$ at 1 M Li^+ concentration [Ref.[3]].

The numerical model assumes galvanostatic (constant current) conditions, i.e., a constant diffusional flux of O_2^- at the carbon surface that is defined by the current density j_a, and a concentration that decays to zero at infinite distance *x* at all times *t*. The Neumann boundary conditions for equation 4 read:

$$D_{\mathrm{O}_2^-}\left(\frac{\partial C_{\mathrm{O}_2^-}}{\partial x}\right)_{x=0} = -\frac{j_a}{N_\mathrm{A} q_{\mathrm{e}-}} \quad \& \quad \left(C_{\mathrm{O}_2^-}\right)_{x\to\infty} = 0\ . \tag{S1}$$

The Li_2O_2 concentration profile is related to the O_2^- concentration profile since the O_2^- concentration determines the Li_2O_2 generation rate according to $1/2\ k^{(2)} C_{\mathrm{Li}^+} C_{\mathrm{O}_2^-}$. The Li^+ concentration is assumed to be constant at 1 M. The Li_2O_2 diffusional flux at the electrode is zero, resulting in the following boundary conditions for equation 4:

$$D_{\mathrm{Li}_2\mathrm{O}_2}\left(\frac{\partial C_{\mathrm{Li}_2\mathrm{O}_2}}{\partial x}\right)_{x=0} = 0 \quad \& \quad \left(C_{\mathrm{Li}_2\mathrm{O}_2}\right)_{x\to\infty} = 0 \tag{S2}$$

We set the Li_2O_2 diffusion coefficient $D_{\mathrm{Li}_2\mathrm{O}_2} = 0.01 \cdot D_{\mathrm{O}_2^-}$, assuming that Li_2O_2 is practically immobile as soon as it nucleates. The model does not specifically account for nucleation and growth of Li_2O_2 crystallites, and it does not distinguish whether supersaturation of $LiO_{2(sol)}$ or $Li_2O_{2(sol)}$ is required to form Li_2O_2 crystals. In terms of diffusion, the model does not account for the increasingly tortuous particulate Li_2O_2 deposit during discharge, i.e., diffusion coefficients remain constant over distance and time. This implies that the actual transport is somewhat worse than the model predicts and that the model is best valid at early stages of discharge.

Nevertheless, the Li_2O_2 concentration profile $C_{\mathrm{Li_2O_2}}(x,t)$ should give a good estimate for the expansion of the particulate Li_2O_2 layer on the electrode surface and the local rate Li_2O_2 will form. A high value of $C_{\mathrm{Li_2O_2}}$ means a large quantity of Li_2O_2 formed at a high rate. We calculate the local Li_2O_2 formation rate $\partial C_{\mathrm{Li_2O_2}}(x,t)/\partial t$ by dividing $C_{\mathrm{Li_2O_2}}(x,t)$ by the timestep Δt. A high local Li_2O_2 formation rate causes high nucleation rates. Li_2O_2 particles will be smaller and more numerous.

To translate local Li_2O_2 formation rates to Li_2O_2 morphologies and Li-O_2 discharge capacities, the following needs to be considered:

(i) Having larger Li_2O_2 particles does not imply higher DISP rates. Considering nucleation and growth theory, larger and less numerous Li_2O_2 particles indicate lower nucleation rates and hence lower DISP rates. This is beneficial in terms of discharge capacities at planar electrodes - in porous carbon electrodes only up to a certain particle size (which is related to the pore size).

(ii) The capacity in porous electrodes is not defined by the size of Li_2O_2 particles, but by the Li_2O_2 pore filling.

We have varied the DISP rate constant $k^{(2)}$, the areal current densities j_a, and the O_2^- diffusion coefficient $D_{O_2^-}$ to cover a the typical range of electrolytes and discharge conditions (Table S5).

Table S2 | Variation of DISP model input parameters

2nd order reaction rate $k^{(2)}$ ($M^{-1}s^{-1}$)	10^1	10^2	10^3
O_2^- diffusion coefficient $D_{O_2^-}$ ($m^2\ s^{-1}$)	10^{-9}	10^{-10}	10^{-11}
Current density j_a (µA cm^{-2})	2	0.2	0.02

The evolution of the O_2^- and Li_2O_2 concentration ($C_{O_2^-}$ and $C_{\mathrm{Li_2O_2}}$) are given as a function of distance x to the planar electrode surface (Figure S6).

Varying the pseudo-first order reaction rate means varying the LiO_2 association rate or the solvation energy of the electrolyte. At a given rate and mobility, an increased DISP rate constant (e.g., 1000 $M^{-1}s^{-1}$ which is in the order experimentally found for MeCN[3]) gives steep O_2^- and Li_2O_2 gradients and high Li_2O_2 concentrations close to the electrode surface (Figure S6a, d). Because of the high reaction rate, the O_2^- concentration profile will soon reach a steady state, whereas the Li_2O_2 concentration steadily increases. Decreasing the DISP rate to moderate (100 $M^{-1}s^{-1}$, Figure S6b, e) or low values (10 $M^{-1}s^{-1}$, Figure S6c, f) leads to flatter concentration gradients, higher O_2^- concentrations and lower local Li_2O_2 concentrations at a given time *t*. A smaller DISP rate also implies that steady-state of the O_2^- concentration profile is reached later and Li_2O_2 formation is generally slower. Considering decreased nucleation rates along with decreased Li_2O_2 formation rates, the Li_2O_2 particle size decreases and the particle number density increases with increasing rate constant. As sketched in Figure S6d-f, Li_2O_2 particles will nucleate faster and closer to the surface at large DISP rates, i.e., in poorly solvating or low DN electrolytes.

The time-dependencies of the surface O_2^- and Li_2O_2 concentrations are given in Figure S6g, h and demonstrate slow Li_2O_2 formation for low DISP rate constants (or low LiO_2 association rates; in high DN electrolytes). Since Li_2O_2 nucleation requires supersaturation of LiO_2 (or Li_2O_2), nucleation rates are low for low DISP rate constants, forming less numerous but large Li_2O_2 particles. Note that the model does not account specifically for Li_2O_2 nucleation and growth and does not distinguish between heterogeneous (surface) and homogenous nucleation rates.

The mutual dependencies of LiO_2 association (DISP) rate constants, species mobility, and applied current density on Li_2O_2 formation rate profiles (and hence Li_2O_2 morphologies) are given in Figure S6i-k. As discussed above, the Li_2O_2 formation rate is highest with high DISP rate constants (fast LiO_2 association), in particular close to the carbon surface. As a consequence, Li_2O_2 particles are largest for

highly dissociating and smallest for poorly dissociation electrolytes. This is in line with SEM measurements shown in Figure S8 and Refs.[5,6].

The O_2^- diffusion coefficient determines the steepness of O_2^- concentration gradients and Li_2O_2 formation rate profiles, and hence Li_2O_2 nucleation and growth rates and Li_2O_2 morphologies, just like the electrolyte's solvation energy and the current density. This has been largely overlooked in recent years. A small diffusion coefficient gives steep O_2^- concentration gradients (Figure S6j) and fast Li_2O_2 formation close to the carbon surface, resulting high near-surface nucleation. A high diffusion coefficient (such as with MeCN), on the other hand, expands shallow concentrations to large distances with concurrent small nucleation rates and relatively large particle sizes, even in poorly dissociating electrolytes. This is in line with capacities and pore fillings given in Figure 1 and Figure 4 (main part).

Increasing the current density raises the O_2^- levels in solution, accelerates Li_2O_2 formation and favours high near-surface nucleation rates (Figure S6k). Concurrently, small particles forms in high density of close to the surface and effectively cover the carbon by a tortuous Li_2O_2 layer of only a few nanometers. The current dependency is in line with SEM and SAXS measurements in Ref. [7] and Refs.[8,9].

These relations explain the unexpectedly high capacities and large Li_2O_2 particles for MeCN electrolytes with high surface area cathodes. They explain how small Li_2O_2 particles (of a few nm) can effectively cover the carbon surface in form of a thin particulate film via solution-mediated disproportionation at conditions previously thought to be prototypical for the surface mechanism. Such effective surface coverage via solution-mediated disproportionation was interpreted as conformal coating formed via consecutive electron transfer (surface mechanism) in the past. Probably, due to limited resolutions with SEM.

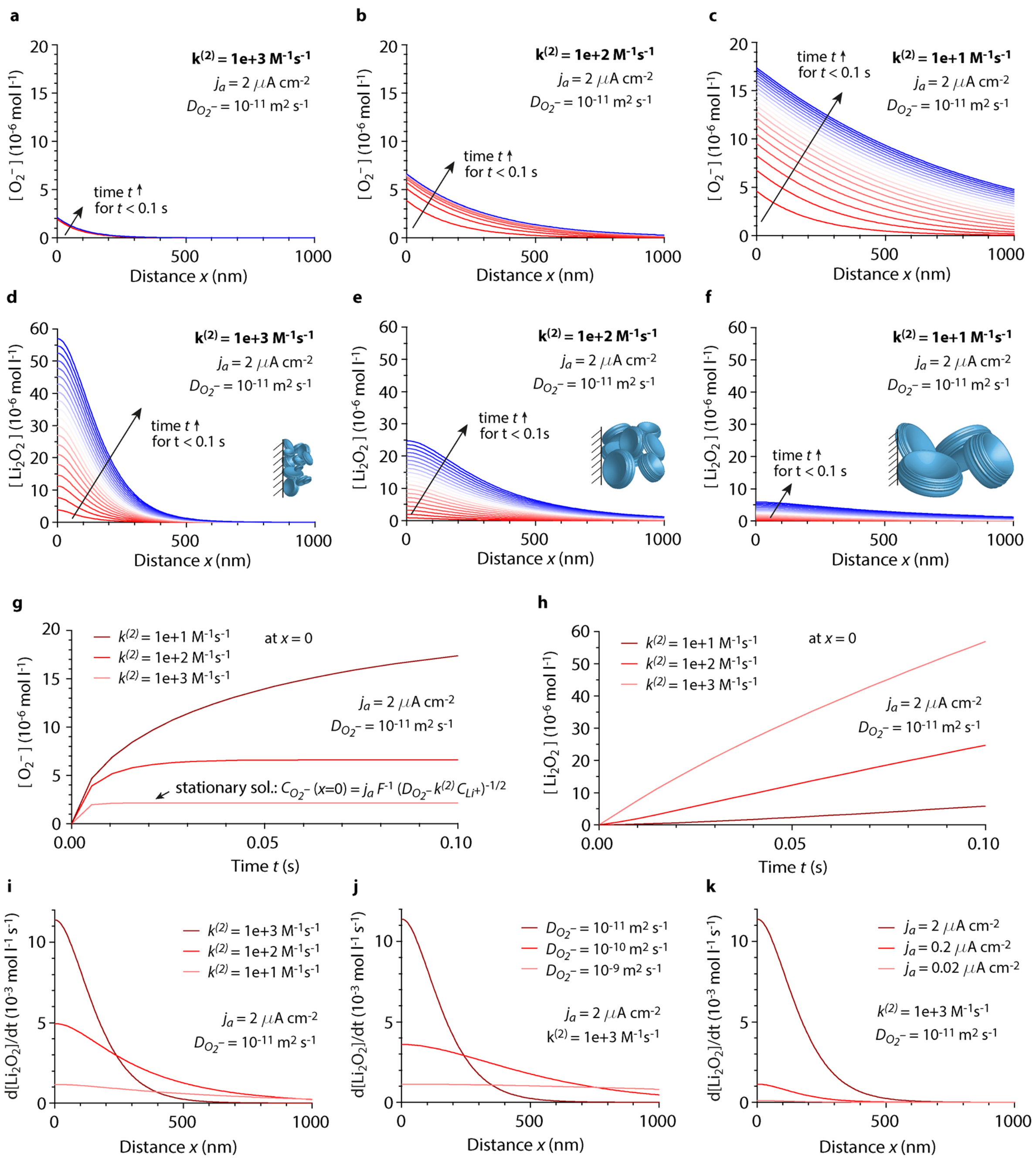

Figure S6. O_2^- and Li_2O_2 concentration profiles derived from the numerical DISP model. O_2^- concentration (a, c, e), and Li_2O_2 concentration profiles (b, d, f) as a function of distance *x* from a planar electrode with increasing time *t* (for $t < 0.1$ s). The current density is set to j_a = 2 μA cm^{-2}, the O_2^- diffusion coefficient to 10^{-11} m^{2}s^{-1} and the reaction rates to $k^{(2)}$ = 1000 M^{-1}s^{-1} (a, b), 100 M^{-1}s^{-1} (c, d), and 10 M^{-1}s^{-1} (e, f). (g)–(h) O_2^- and Li_2O_2 concentrations at $x = 0$ as a function of time *t* for three different reaction rates ($k^{(2)}$ = 1000 M^{-1}s^{-1}, 100 M^{-1}s^{-1} and 10 M^{-1}s^{-1}). (i)–(k) Li_2O_2 formation rate profiles at $t = 0.1$ s, at varying rate constants $k^{(2)}$ (i), O_2^- diffusion coefficients $D_{O_2^-}$ (j), and current densities j_a (k).

Supplementary Note 3: Degree of dissociation dependency in DME electrolytes

To independently validate capacity and particle morphology dependencies on the degree of dissociation and current densities, we performed galvanostatic discharge, electron microscopy and rotating ring disc electrode (RRDE) using electrolytes with DME as solvent. Based on its low solvation energy, DME poorly dissociates $LiO_{2(sol)}$, similar to MeCN. We used 1 M LiTFSI in DME, 0.9 M LiTFSI + 0.1 M $LiNO_3$ in DME and 0.5 M LiTFSI + 0.5 M $LiNO_3$ in DME as electrolytes to test the dependency of the Li_2O_2 morphology and capacity on the degree of dissociation. Adding NO_3^- significantly increases the concentration of dissociated $LiO_{2(sol)}$, but does not primarily affect Li^+, O_2 and $LiO_{2(sol)}$ diffusion coefficients.

Specific capacities for the three electrolytes in GC, SP and KB cathodes draw a clear picture (Figure S7). With increasing areal current density, the capacity gain in the NO_3^- containing electrolytes increases. This is in line with our ORR model as illustrated by the numerical simulation (Supplementary Note 2). Large Li_2O_2 particle sizes (large characteristic lengths δ) and high capacities can be well achieved in poorly dissociating electrolytes, if the current density is low (see Eq. S1). Note that the hypothetical conformal coating thickness on the GC electrode corresponds to 2.05, 2.7 and 8.93 nm for the 1/0, 0.9/0.1 and 0.5/0.5 LiTFSI/$LiNO_3$ ratio, respectively (Figure S7a).

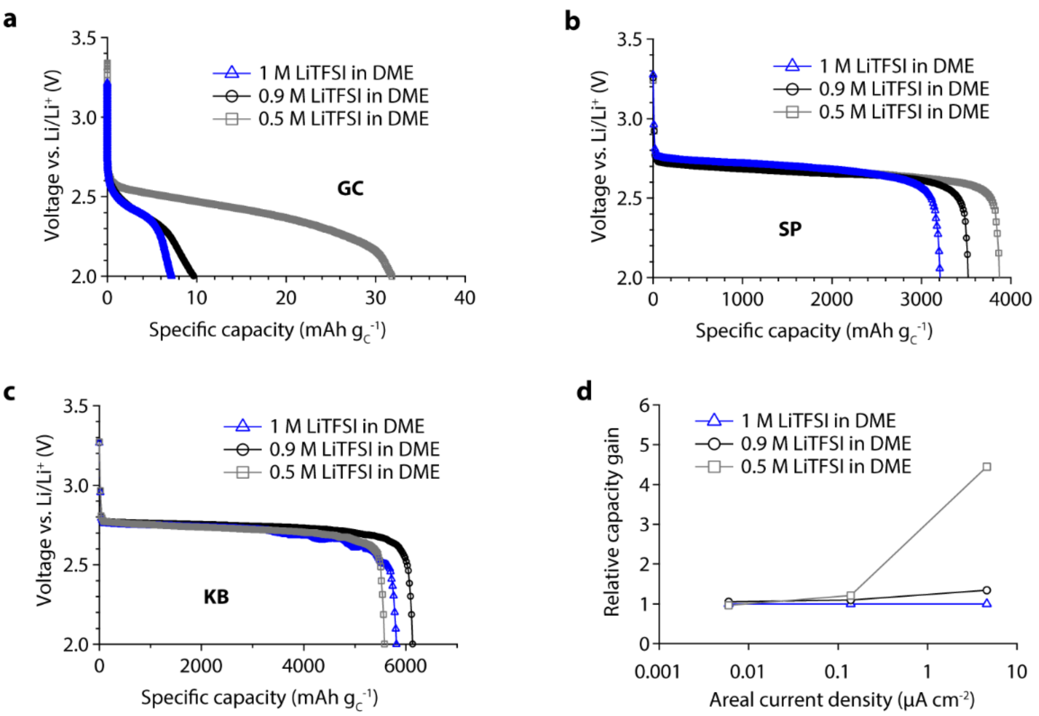

Figure S7. Galvanostatic discharge with DME and GC, SP, KB cathodes at 180 µA cm_{geom}^{-2}. (a) Full galvanostatic discharge with 1 M LiTFSI / DME, 0.9 M LiTFSI + 0.1 M $LiNO_3$ /DME, 0.5 M LiTFSI + 0.5 M $LiNO_3$ /DME electrolytes, and GC cathodes (4.6 µA cm_{real}^{-2}). (b) Full galvanostatic discharge with the same electrolytes and SP cathodes (0.14 µA cm_{real}^{-2}). (c) Full galvanostatic discharge with the same electrolytes and KB cathodes (0.006 µA cm_{real}^{-2}). (d) Relative capacity gain with respect to the 1M LiTFSI / DME electrolyte as a function of areal current density (µA cm_{real}^{-2}).

According to previous understanding, the 1 M LiTFSI/DME electrolyte in combination with high current densities (low surface area carbons such as GC) should be most prototypical for the surface mechanism (conformal Li_2O_2 coating via consecutive electron transfer). However, particles are evident with all salts as seen by SEM (Figure S8). In accord with our ORR model, the particle size increases with increasing degree of LiO_2 dissociation (increasing NO_3^- concentration), following the discharge capacities in Figure S8f. Clearly, the Li_2O_2 morphology for 1 M LiTFSI/DME is particulate, with small “toroidal” particles in

the order of 20-40 nm (see detail Figure S8e). This rejects surface discharge and implies solution discharge via LiO_2 disproportionation, even at extremely high current densities and in low donor number electrolytes. The hypothetical conformal coating thickness (of 4.28 nm, estimated from discharge capacities and carbon BET area) cannot be used as an indicator for discriminating between surface or solution mechanism.

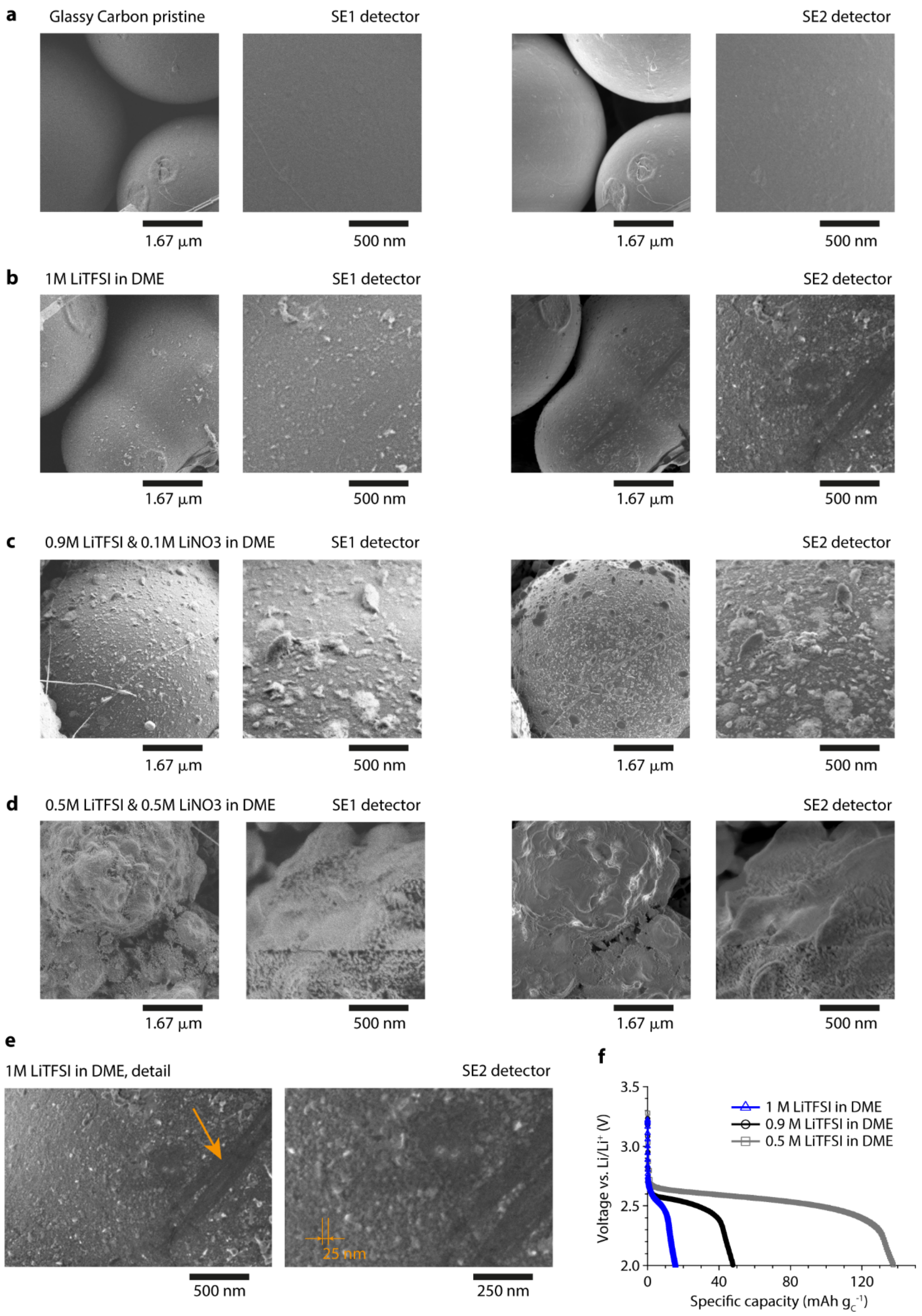

Figure S8. Scanning electron microscopy (SEM) – Galvanostatic discharge with DME / GC. SEM images of two secondary electron detectors (SE1 and SE2) of pristine (a) and discharged (b–d) GC cathodes at 50 µA cm_{geom}^{-2} (= 1.3 µA cm_{real}^{-2}). 1 M LiTFSI / DME (b), 0.9 M LiTFSI + 0.1 M $LiNO_3$ /DME (c), and 0.5 M LiTFSI + 0.5 M $LiNO_3$ /DME (d) were used as electrolytes. A detailed view of the 1 M LiTFSI / DME image (e) indicates the particulate Li_2O_2 morphology on the GC surface. Most Li_2O_2 particles are > 25 nm, which is incompatible with the ORR surface mechanism. The arrow indicates a region of pristine GC surface, initially covered by a glass fibre of the Whatman separator during discharge. (f) Discharge curves of the samples in (b–e).

Supplementary Note 4: Degree of pore filling and electrode swelling

The high degrees of pore filling in high surface area cathodes (SP and KB, see Fig. 4 and Table S1) can only be explained by significant electrode swelling to pack the full volume of Li_2O_2 into the cavities of the KB electrode. For MeCN the thickness of the dry carbon electrode after full discharge increased by roughly 80% (measured by a micrometer screw). 80% electrode swelling corresponds to a final Li_2O_2 pore occupation of roughly 55%. To generate a large mechanical stress via Li_2O_2 crystallization the contact angle between Li_2O_2 phase and carbon surface need to be larger than 90° (see Ref. [10]). Exclusive Li_2O_2 surface growth would engulf carbon black particles without significant mechanical deformation.

Supplementary Note 5: Rate performance with MeCN vs. TEGDME/H_2O electrolyte

As we show with the numerical DISP model, the Li_2O_2 particle size is a trade-off between three parameters, the LiO_2 association (DISP) rate, which is related to (i) solvation energy (electrolyte DN), (ii) the true areal current density, and (iii) the superoxide mobility, as they determine the O_2^- and Li_2O_2 concentration profile normal to the electrode surface.

The correlation "the larger the Li_2O_2 particle size – the larger the capacity" is only true for planar electrodes. In porous carbon electrodes (e.g., carbon black electrodes) not only the particle size, but primarily the pore filling determines the capacity.

An additional aspect related to species mobilities and capacities, is the O_2 mass transport across free-standing electrodes. At very high rates, O_2 mass transport limits the local current density, resulting in a gradient of Li_2O_2 particle sizes across the electrode thickness. A high O_2 mobility allows for a higher capacity, as this guarantees a more uniform and high degree of Li_2O_2 pore filling.

One of the main outputs of this work is that sudden cell death at the end of discharge is not caused by electron transport limitation (passivation via a surface film) but by mass transport limitation of the reactive species (O_2, O_2^-, LiO_2, Li^+) in the increasingly tortuous network of Li_2O_2/carbon particles. Hence, improved species mobility would also lower the kinetic overpotential for a given carbon/Li_2O_2 morphology, which increases discharge capacities.

Galvanostatic discharge at high rates and potentiostatic discharge for two different systems (KB electrode with 1 M LiTFSI/MeCN or 1 M LiTFSI in TEGDME with 4000 ppm H_2O) are given in Figure S9. Please, compare the very high geometrical current densities with standard current densities used in literature (Figure S1). The MeCN electrolyte outperforms TEGDME with 4000 ppm H_2O, specifically at high rates.

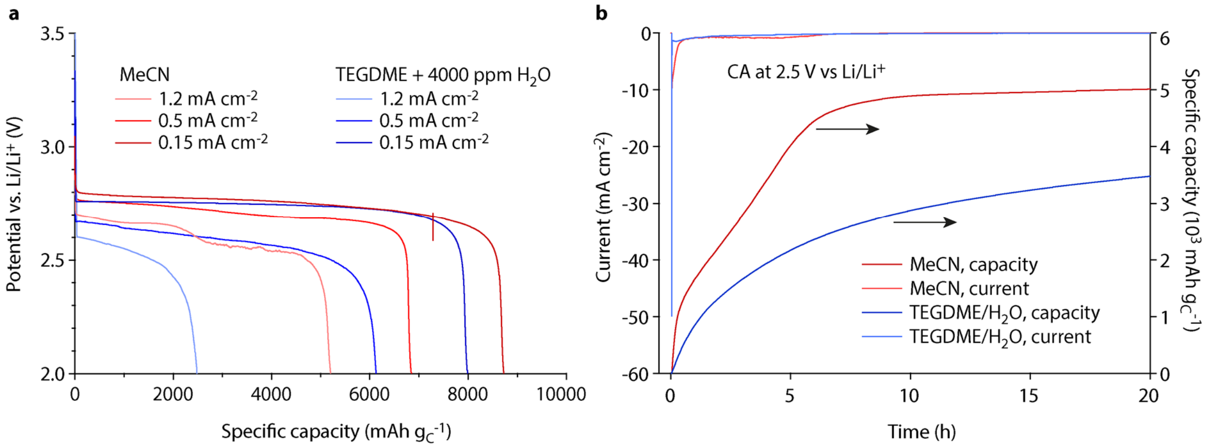

Figure S9. Galvanostatic discharge at different rates and potentiostatic discharge. (a) Galvanostatic discharge at 1.2, 0.5 and 0.15 mA cm^{-2} (currents normalized by the electrode area, 0.5 cm^2) for a KB cathode and 1 M LiTFSI in MeCN or 1 M LiTFSI in TEGDME/H_2O. (b) Current (normalized by the electrode area, 0.5 cm^2) vs. time and specific capacity versus time for potentiostatic discharge at 2.5 V vs. Li/Li^+ for the same two systems.

5. Supplementary References

1 Viswanathan, V. *et al.* Electrical conductivity in Li2O2 and its role in determining capacity limitations in non-aqueous Li-O2 batteries. *The Journal of Chemical Physics* **135**, 214704, (2011).

2 Zhang, Y. *et al.* Potential-Dependent Generation of O2– and LiO2 and Their Critical Roles in O2 Reduction to Li2O2 in Aprotic Li–O2 Batteries. *The Journal of Physical Chemistry C* **120**, 3690-3698, (2016).

3 Zhang, Y. *et al.* Amorphous Li2O2: Chemical Synthesis and Electrochemical Properties. *Angewandte Chemie International Edition* **55**, 10717-10721, (2016).

4 Compton, R. G., Laborda, E. & Ward, K. R. in *Understanding voltammetry: simulation of electrode processes* Ch. Second-Order Chemical Kinetic Mechanisms, 123-143 (Imperial College Press, 2014).

5 Burke, C. M., Pande, V., Khetan, A., Viswanathan, V. & McCloskey, B. D. Enhancing electrochemical intermediate solvation through electrolyte anion selection to increase nonaqueous Li-O2 battery capacity. *Proceedings of the National Academy of Sciences of the United States of America* **112**, 9293-9298, (2015).

6 Aetukuri, N. B. *et al.* Solvating additives drive solution-mediated electrochemistry and enhance toroid growth in non-aqueous Li–O2 batteries. *Nature Chemistry* **7**, 50, (2014).

7 Prehal, C. *et al.* In situ small angle X-ray scattering reveals solution phase discharge of Li-O2 batteries with weakly solvating electrolytes. *PNAS* **118**, e2021893118, (2021).

8 Adams, B. D. *et al.* Current density dependence of peroxide formation in the Li–O2 battery and its effect on charge. *Energy & Environmental Science* **6**, 1772-1778, (2013).

9 Griffith, L. D., Sleightholme, A. E. S., Mansfield, J. F., Siegel, D. J. & Monroe, C. W. Correlating Li/O2 Cell Capacity and Product Morphology with Discharge Current. *ACS Applied Materials & Interfaces* **7**, 7670-7678, (2015).

10 Scherer, G. W. Stress from crystallization of salt. *Cem. Concr. Res.* **34**, 1613-1624, (2004).
